# Protocol-based Surgical Intervention to Manage Ventricular Septal Rupture from a Tier Two City

**DOI:** 10.21470/1678-9741-2020-0652

**Published:** 2023

**Authors:** Riju Nair, Kumar Subbaiyan, Krishnan Rm, Rajan Mani, Balamurugan Kathamuthu

**Affiliations:** 1 Department of Cardiothoracic and Vascular Surgery, Meenakshi Mission Hospital and Research Center, Madurai, Tamil Nadu, India

**Keywords:** Myocardial Infarction, Ventricular Function, Left, Ventricular Septal Rupture, Renal Insufficiency, Shock, Cardiogenic, Risk

## Abstract

**Introduction:**

This study analyzes the outcome of a protocol-based surgical approach for ventricular septal rupture (VSR). The study also clarifies the appropriate time for intervention.

**Methods:**

This is a single-center retrospective analysis of all VSR cases evaluated between February 2006 and March 2020. Cases were managed using the same protocol. Patients were divided into two cohorts - early (those in whom our protocol was instituted within 24 hours of diagnosis) and delayed (intervention between 24 hours and seven days after diagnosis). All-cause mortality was considered as the outcome.

**Results:**

The mean age of presentation was 60.1 years, and 75.9% of the patients were men. Cardiogenic shock was the most common mode of presentation. Our analysis validates that once a patient develops VSR, age, sex, comorbidities, left ventricular function, and renal failure at the time of presentation do not have a statistically significant impact on the outcome. The sole factor to have an impact on the outcome was time of intervention. All patients in the delayed cohort expired after surgery, which dragged the overall mortality to 34.5%, whereas 95% of patients in the early cohort are still on follow-up. The mortality in this group was 5% (P≤0.001).

**Conclusion:**

Early surgical intervention has proven benefits over delayed approach. Surgical intervention in the early part of the disease reduces the risk and thus improves the outcome. The extreme rarity makes VSR an uncommon entity among surgeons. A protocol-based approach makes the team adapt to this unfamiliar situation better.

## INTRODUCTION

Mechanical complications aggravating acute myocardial infarction (AMI) have always been a cause for concern for surgeons worldwide. Even though the advent of cardiac catheterization has improved the outcome for AMI significantly, survival once mechanical complication sets in still remains poor^[1]^. In a tier two city, where the luxuries of portable diagnostics and advanced cardiac care are limited, diagnosis of any mechanical complication is usually a death sentence. Lack of authentic protocol to manage these complications further adds on to the agony. Early suspicion, timely diagnosis, and prompt intervention remain the key for patient survival.

Clinically, ventricular septal rupture (VSR) is the most encountered form of mechanical complication. Aggressive early reperfusion strategies have reduced the incidence of VSR from 1% - 2% to about 0.17% - 0.31%^[2]^. However, once developed, prognosis is dismal^[3]^. Nonsurgical options for managing VSR are associated with dire consequences. Though the scientific community agrees on surgical repair as the best available modality to address this issue^[4]^, the “apposite time” to intervene has always been a topic of debate.

The original description of the most popular infarct exclusion technique by David et al.^[5]^ reported excellent results with early surgical intervention. Surgical mortality in the series reported by them was only 19%. Our extensive search for supporting evidence lead us to studies by Arnaoutakis et al.^[6]^ and Coskun et al.^[7]^. Arnaoutakis et al.^[6]^ have probably published the largest series till date and advocate a delayed surgical approach. However, the study did not consider those patients who expired while waiting for surgery. Coskun et al.^[7]^ reported higher mortality in patients operated early, but the authors themselves declare that patients in that specific cohort were already in intractable cardiogenic shock before surgery. These studies were based on the notion that surgical intervention on mature myocardial tissue was associated with improved surgical outcome. Multiple single-center experiences in favor of early surgical intervention were also analyzed. Patients who develop VSR generally progress to low cardiac output, which usually worsens with time. This increases the risk of intervention significantly. Early surgical intervention arrests the clinical decline in these patients and this concept forms the crux for studies advocating early intervention. A study comparing intervention at two different times do not exist.

After careful consideration of the abovementioned studies, we, at Meenakshi Mission Hospital, have retrospectively analyzed cases presented to us during the past one and a half decade. All patients were managed using the same protocol devised by us specifically to address VSR. Case records were analyzed, and patients were followed up. The sole purpose of our analysis was to analyze all possibilities and determine an appropriate time for intervention.

## METHODS

We retrospectively analyzed cases presented to us during the past 15 years (from February 2006 to March 2020). A total of 32 VSR patients were evaluated by us all through this period. Our protocol to manage VSR is a three-step strategy which was offered to all.

The strategy includes:

1. Emergency minimal shots (three shots) - coronary angiogram. The advantage of dedicated and fixed protocol-based angiogram is that it reduces time for the procedure significantly and also reduces the burden of contrast on an already compromised kidney.
2. Intra-aortic balloon pump (IABP) insertion.
  We advocate IABP insertion for all patients irrespective of the hemodynamic status. Insertion of IABP not only maintains peripheral perfusion but also reduces afterload on the left ventricle, thereby reducing shunt fraction. Moreover, patients with mechanical complication can become unstable and deteriorate rapidly^[8]^, and IABP helps to tide over this crisis until a definitive extracorporeal circulation is established. Anecdotal evidence of these patients crashing immediately after induction also favors IABP insertion preoperatively.
3. Emergency double patch surgical repair using David’s infarct exclusion technique^[5]^.
  The role of early surgical intervention will be clearly explained later. A detailed description of the surgical procedure is portrayed below.

Anesthesia

General anesthesia was induced with thiopentone (5 mg/kg), fentanyl (5 mcg/kg), and midazolam (25 mcg/kg) and was maintained with vecuronium, propofol, isoflurane, oxygen (O₂), and medical air mixture. Bispectoral index was used to monitor depth of anesthesia. The main anesthetic concern was to reduce left ventricular (LV) afterload while maintaining perfusion pressures. Reducing LV afterload reduces tension on suture lines. Maintaining systemic vascular resistance (SVR)/pulmonary vascular resistance (PVR) ratio is also important as it reduces the shunt fraction. This was achieved with nitroglycerine and dobutamine infusions, which maintain systemic blood pressure without increasing SVR. Patients are then ventilated with air - O₂ mixture (FiO₂ - 50%) to maintain normocarbia and prevent hyperoxia. This further helps to maintain SVR/PVR ratio.

### Perfusion

Conventional ultrafiltration technique with haemofilter was used to prevent volume overload. Conduct of cardiopulmonary bypass (CPB) is otherwise the same as with any case of open-heart surgery.

### Surgery

All cases were approached through a median sternotomy. Intraoperative transesophageal echocardiogram was used to assess ventricular function and check adequacy of repair. CPB was established through aortic bicaval cannulation and was conducted in moderate hypothermia. del Nido cold blood cardioplegia infused at 12 °C ensured adequate myocardial protection. In cases where a concomitant coronary artery bypass grafting (CABG) was also performed, distal anastomosis was constructed first for better myocardial protection. A transinfarct ventriculotomy, 1 - 2 cm lateral and parallel to the left anterior descending artery (LAD), usually gives an excellent exposure. LAD is salvaged as far as possible. We do not advocate doing an infarctectomy and follow a modified version of the infarct exclusion technique originally described by David et al.^[5]^. An additional small autologous pericardial patch is used on the right ventricular side along with larger Dacron patch in the LV side to repair the defect. The Dacron patch on the LV side is larger and usually extends till the ventriculotomy margin to exclude the infracted myocardium from the high-pressure ventricular cavity. The two patches were sutured with interrupted nonabsorbable polypropylene (4-0) sutures in a way that the edges of the defect get sandwiched between the two patches. Sutures are taken well away from the margins of the defect on healthy myocardium. The ventriculotomy is then closed with nonabsorbable polypropylene (4-0) sutures in an interrupted manner, followed by continuous manner, with Teflon strips on either side.

This protocol was followed in all patients irrespective of the hemodynamic status and time of presentation. Universal early institution of this protocol makes the study unique from others on the same topic. Out of the 32 patents, three did not consent for surgery and hence were excluded from the study. All the three patients developed intractable cardiogenic shock and succumbed to their illness. The remaining cases were considered for analysis. Our approach towards a case of VSR was to institute our protocol at the earliest time, but this was delayed in some in account of late referral. Cases were divided into two groups - one in whom our protocol was instituted early (*i.e.*, within 24 hours of diagnosing VSR) and other in whom intervention was delayed (*i.e.*, between 24 hours and seven days after diagnosis). All-cause mortality was considered as the outcome. The two cohorts were matched for age, sex, comorbidities, LV function, and renal failure at the time of presentation (Table 1). These variables were chosen as they showed statistically significant impact on the outcome in studies done in the past. Data were presented as counts and percentage for categorical data and mean for continuous variables. Comparison was done using Pearson’s Chi-square test and Fisher’s *t*-test as appropriate. A *P*-value < 0.05 was considered significant. The observations made were statistically analyzed for results.

**Table 1:** Two cohorts matched for age, sex, comorbidities, left ventricular function, and renal failure.

Parameter			Time of Intervention				*P*-value
			Early		Delayed		
			Cases	%	Cases	%	
Sex		Male	15	68.2	7	31.8	0.6
		Female	5	71.4	2	28.6	NS
Age		≤ 60 years	11	73.3	4	26.7	0.59
		≥ 61 years	9	64.3	5	35.7	NS
	Type 2 diabetes mellitus	Yes	14	77.8	4	22.2	0.23
		No	6	54.5	5	45.5	NS
	Hypertension	Yes	6	66.7	3	33.3	0.59
		No	14	70	6	30	NS
LV function (at presentation)		Good LV function	1	50	1	50	0.40
		Mild LV dysfunction	2	100	0	0	NS
		Moderate LV dysfunction	6	54.5	5	45.5	
		Severe LV dysfunction	11	78.6	3	21.4	
Renal failure (at presentation)		Yes	11	64.7	6	35.3	0.69
		No	9	75	3	25	NS

Good LV function=ejection fraction (EF) ≥ 50%; mild LV dysfunction=EF ≥ 40 but < 50%; moderate LV dysfunction=EF ≥ 35 but < 40%; severe LV dysfunction=EF < 35%

LV=left ventricular; NS=not significant

## RESULTS

A total of 29 patients were managed using our protocol during the study period. Table 2 depicts the demographic profile of patients included in the study. The mean age of the entire cohort at the time of presentation was 60.1 years (range: 42 - 70 years); 75.9% of the patients were men (22 of 29). Cardiogenic shock was the most common mode of presentation (62.1% [18 of 29]) followed by pulmonary edema and chest pain.

**Table 2:** Patients’ demographic profile (n = 29).

Factor		Number: n (%)
Age	≤ 60	15 (51.7%)
	≥ 61	14 (48.3%)
Sex	Male	22 (75.9%)
	Female	7 (24.1%)
Comorbidities	Diabetes mellitus	18 (62.1%)
	Systemic hypertension	9 (31.1%)
Presentation	Cardiogenic shock	18 (62.1%)
	Pulmonary oedema	6 (20.7%)
	Chest pain	5 (17.2%)
Location of VSR	Apical	27 (93.1%)
	Muscular	2 (6.9%)
Time of presentation	Early	20 (68.9%)
	Delayed	9 (31.1%)
Renal failure (at presentation)	Yes	17 (58.6%)
	No	12 (41.4%)
Associated procedure	Yes	16 (55.2%)
	No	13 (44.8%)
CABG	Single vessel	12 (75%)
	Multivessel	4 (25%)
	LAD	8 (66.7%)
	OM	3 (25%)
	RCA	1 (8.3%)
	OM & RCA	1 (25%)
	LAD & OM	2 (50%)
	LAD & RCA	1 (25%)

CABG=coronary artery bypass grafting; LAD=left anterior descending artery; OM=obtuse marginal artery; RCA=right coronary artery; VSR=ventricular septal rupture

Presence or absence of comorbidities (diabetes mellitus and systemic hypertension) did not seem to have significant impact on the outcome (*P*=0.59 and *P*=1.0, respectively). Seventeen (58.6%) patients were already in acute renal failure before intervention, but this again did not have a statistically significant impact on mortality (*P*=0.6). One of these patients was a renal transplant recipient who in view of early surgical intervention had good surgical outcome and is on follow-up. Sixteen (55.2%) patients underwent CABG along with VSR repair. Most of them (75% [12 of 16]) had single-vessel bypass graft surgery. We did multivessel CABG along with VSR repair in four patients. LAD was the most commonly grafted vessel followed by obtuse marginal artery and right coronary artery. However, addition of CABG to VSR repair did not demonstrate a statistically significant impact on the outcome (*P*=0.8). The LV function was assessed with bidimensional echocardiography using the biplane Simpson’s method. Fourteen (48.2%) patients already had severe LV dysfunction at the time of intervention of which 10 (71.4%) are doing well on follow-up. Eleven (37.9%) patients were diagnosed with moderate LV dysfunction and two (6.9%) patients with normal LV function. Six out of the eleven patients (54.5%) and one out of the two patients (50.0%) are on follow-up. Two (6.9%) patients who had mild LV dysfunction at the time of presentation are both on follow-up. Surprisingly, LV function at the time of intervention did not have a significant impact on survival (*P*=0.87).

The mean follow-up period was 168.96 months (14.01 years) (range: 176 months - 6 months). All patients who are in follow-up are either in New York Heart Association class I or II symptoms. Fatigue is the most common symptom expressed. Follow-up echocardiogram showed no worsening of cardiac status and hence all patients were managed symptomatically.

The overall mortality during the study period was 34.5% (10 of 29 patients). Table 3 compares the outcome between the two cohorts. Further analyses of our results reveal that mortality was higher in the delayed cohort. All patients in the delayed cohort succumbed to their illness even after repair. Most of the delayed cohort (7 of 9 patients [77.8%]) developed low cardiac output syndrome in the immediate postoperative period and could not be saved. One patient had worsening of renal status and could not be saved in spite of renal replacement therapy. The last patient in the cohort developed patch dehiscence in the second postoperative day and was taken for revision surgery. Even though he had a stormy postoperative course after revision, he was discharged in a stable condition on the 16^th^ postoperative day. However, he was found unconscious and declared dead at home after two months. On the contrary, 19 of 20 patients (95.0%) in the early cohort are still on follow-up. One patient in this group developed multiorgan dysfunction secondary to urosepsis and could not be salvaged in spite of maximal supportive therapy. The mortality in this group of the study is 5.0%. This strikingly significant difference was also reflected statistically (*P*-value ≤ 0.001).

**Table 3:** Outcome and subgroup analysis.

Outcome	Early (n=20)	Delayed (n=9)		*P*-value
Mortality	1 (5%)	9 (100%)		< 0.001
On follow-up	19 (95%)	0 (0%)		Significant
*Subgroup Analysis (mortality n=10)*				
Mortality	Early (n=1)		Delayed (n=9)	
Early mortality	1 (100%)		8 (88.9%)	
Delayed mortality	0 (0%)		1 (11.1%)	

Early mortality=mortality within 30 days after surgery; delayed mortality=mortality beyond 30 days of surgery

Patch dehiscence secondary to friable myocardial tissue was encountered only in one patient in the delayed cohort. None of the patients in the early cohort had this issue, and the authors of this study believe that intervening in the early part of the disease alleviates this problem.

Studies also comment about short-term and long-term survival after surgery. Subgroup analyses of the overall study (Table 3) reveal that all mortality except for one (9 of 10 patients [90%]) occurred in the early postoperative period, *i.e.*, within 30 days after surgery. We only had one patient who survived beyond 30 days and expired two months after surgery. The vast difference in numbers makes the concept of short-term and long-term survival irrelevant in the present context.

## DISCUSSION

Ever since the first report of surgical repair of VSR by Cooley et al. in 1957^[9]^, many surgeons have attempted to develop techniques to address this issue. For an entity which has a reported incidence of < 1% and mortality > 50%, developing an authentic protocol was difficult. A breakthrough was reported by Daggett et al. in 1977, where he described a transinfarct incision and repair replacing the necrotic muscle. Techniques including infarctectomy and LV and septal restoration/reconstruction were also popular during this period, but the operative mortality still remained high. It was in 1987 that David et al.^[5]^ described the first infarct exclusion technique which not only eliminates interventricular shunt but also prevents ventricular remodeling and aneurysm formation. This additional benefit might have been the reason for a low operative mortality in his series.

**Fig. 1:**
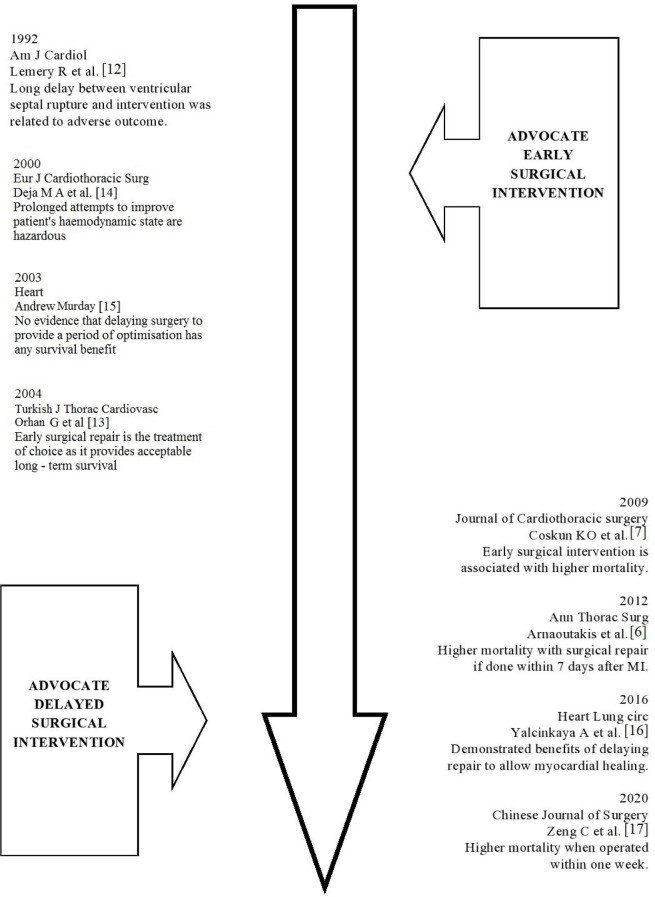
Timeline of various studies and their inference. MI=myocardial infarction.

Subsequently, multiple attempts have been made to better understand the management of VSR. Though initial studies were in favor of early surgical intervention, somewhere in history the concept of delayed surgical approach gained popularity (Figure 1). The propagators of this approach believe that an elective surgical repair after sufficient scar formation is associated with better outcome. But these studies completely “ignore” the subset of patients who die or slip into intractable cardiogenic shock while waiting for surgery. Even though repair on a fibrosed myocardium is technically easier compared to an acutely inflamed myocardium, the authors of the present study strongly believe that this should never be a reason to put the patients to trial. It is because of this major glitch that these strategies were aptly labeled as a technique of “unnatural selection” by Honey et al.^[10]^.

One of the largest available series on VSR is by Arnaoutakis et al.^[6]^, where they establish higher mortality if the patient was operated early. Overall operative mortality in their series was 42.9%. The figures rose to 54.1% when the surgery was conducted within seven days from myocardial infarction and fell drastically to 18.4% when surgery was performed after seven days. Multivariate logistic regression to find the relation between operative mortality and time of intervention demonstrate increased odds of mortality with shorter time intervals. However, this is a retrospective analysis of data from the Society of Thoracic Surgeons (STS) Database and clarity regarding hemodynamic status of those intervened early and time between diagnoses of VSR and surgical intervention is lacking. Lack of data regarding those who died waiting for surgery is also a limitation of that study.

Our study agrees with similar studies in terms of age at presentation. The mean age of presentation in our study was 60.1 years, with a male preponderance. We did not find any significant association between mortality and other variables as demonstrated in other studies. It was observed on literature review that renal failure was quoted as having an adverse impact on survival. Surprisingly, even though 58.6% (17 of 29 patients) of our patients had renal failure at the time of intervention, it did not seem to impact survival. Another study reports LV function to be an important determinant of outcome. Conversely, even though 48.3% (14 of 29 patients) were already diagnosed with severe LV dysfunction before surgery, this again did not appear to affect the outcome. Underestimation of the LV function in an acute setting of stunned/hibernating myocardium might be the reason for this disparity. Location of VSR is yet another variable which was closely associated with outcome. Nakajima et al.^[11]^ reported higher mortality among posterior VSR compared to anterior VSR. Ninety-three percent of patients in the present study (27 of 29 patients) had apical VSR, and the remaining two had muscular VSR. The disproportionate distribution of cases among the two categories in the present study might be a reason why our statistical analysis did not reveal any significance. Hence, the authors of the present study advice to view this observation with caution. Comorbidities like diabetes mellitus and systemic hypertension though closely related to coronary artery disease had little impact on the outcome of patients who underwent surgical repair of VSR. Concomitant CABG along with VSR is another area of debate. Our study is in accord with the majority who prove that addition of CABG did not have any survival benefit. Most cases in our cohort had single-vessel disease, and LAD was the most commonly grafted vessel.

The crux of our study is its mortality and mean follow-up period. The mean follow-up period is 14.01 years, which is probably one of the largest available in literature. This emphasizes the better long-term outcome with early surgical intervention. Overall mortality in our study, which includes both early and late intervention, was 34.5%. One of the earlier reported series, the GUSTO 1 trail, presented a surgical mortality close to 47%. The recent series from the STS Database furnish an operative mortality of 42.9%. The relatively low mortality in our series is clearly the result of our early intervention protocol. The exceptionally low mortality of 5% with an extended follow-up period of 14.1 years in the cohort where intervention was within 24 hours further emphasize the favorable short-term and long-term outcomes coupled with our protocol.

Limitations

The main limitation of the present study is its retrospective design. Though thoroughly scrutinized, the potential for selection bias cannot be overlooked as it is being performed in a single center. Not randomizing patients to the two cohorts makes the study “statistically weak”, but for an entity known for its rarity and mortality, prospects of clinical randomization are virtually nonexistent.

## CONCLUSION

VSR is a rare but dreadful complication of AMI. In the contemporary era where portable diagnostics have become more accessible, it is not unusual to encounter VSR in routine practice. Catheter-based intervention has been described but not without pitfalls. Emergency surgical intervention appears to be the only reasonable treatment option that can be offered to patients. The extreme rarity of this entity together with lack of clarity regarding its management makes it an uncommon surgical nightmare for most surgeons. Nonetheless, this should not be a reason to deny the benefits of early surgical intervention. Early surgical intervention has proven short-term and long-term benefits over the “unnatural selection” technique. IABP has demonstrated to have an exemplary role to tide over the initial crisis. A protocol-based early surgical approach makes the surgical crew adapt to this unfamiliar situation better. More studies which validate the benefits of early surgical intervention are the need of the hour.
